# Glucocorticoid sensitivity in Behçet's disease

**DOI:** 10.1530/EC-12-0056

**Published:** 2012-10-24

**Authors:** R A M Quax, J A M van Laar, R van Heerebeek, K Greiner, E Ben-Chetrit, M Stanford, G R Wallace, F Fortune, M Ghabra, M Soylu, J M W Hazes, S W J Lamberts, J H Kappen, P M van Hagen, J W Koper, R A Feelders

**Affiliations:** 1 Department of Internal Medicine, Erasmus MC University Medical Center 's-Gravendijkwal 2303015 CE, Rotterdam The Netherlands; 2 Department of Immunology, Erasmus MC University Medical Center 's-Gravendijkwal 2303015 CE, Rotterdam The Netherlands; 3 St John's Hospital Jerusalem Israel; 4 Department of Medicine Hadassah-Hebrew University Medical Center Jerusalem Israel; 5 Department of Ophthalmology King's College London UK; 6 Academic Unit of Ophthalmology University of Birmingham Birmingham UK; 7 Department of Oral Medicine Queen Mary's College London UK; 8 University Hospital Damascus Syria; 9 Department of Ophthalmology University of Cukurova School of Medicine Adana Turkey; 10 Department of Rheumatology, Erasmus MC University Medical Center RotterdamThe Netherlands

**Keywords:** Behçet's syndrome, single nucleotide polymorphism, glucocorticoid sensitivity

## Abstract

**Objective:**

Glucocorticoid (GC) sensitivity is highly variable among individuals and has been associated with susceptibility to develop (auto-)inflammatory disorders. The purpose of the study was to assess GC sensitivity in Behçet's disease (BD) by studying the distribution of four GC receptor (GR) gene polymorphisms and by measuring *in vitro* cellular GC sensitivity.

**Methods:**

Healthy controls and patients with BD in three independent cohorts were genotyped for four functional GR gene polymorphisms. To gain insight into functional differences in *in vitro* GC sensitivity, 19 patients with BD were studied using two bioassays and a whole-cell dexamethasone-binding assay. Finally, mRNA expression levels of GR splice variants (GR-α and GR-β) were measured.

**Results:**

Healthy controls and BD patients in the three separate cohorts had similar distributions of the four GR polymorphisms. The Bcll and 9β minor alleles frequency differed significantly between Caucasians and Mideast and Turkish individuals.

At the functional level, a decreased *in vitro* cellular GC sensitivity was observed. GR number in peripheral blood mononuclear cells was higher in BD compared with controls. The ratio of GR-α/GR-β mRNA expression levels was significantly lower in BD.

**Conclusions:**

Polymorphisms in the GR gene are not associated with susceptibility to BD. However, *in vitro* cellular GC sensitivity is decreased in BD, possibly mediated by a relative higher expression of the dominant negative GR-β splice variant. This decreased *in vitro* GC sensitivity might play an as yet unidentified role in the pathophysiology of BD.

## Introduction

Behçet's disease (BD) is an inflammatory disorder characterized by recurrent episodes of orogenital ulcers, uveitis, arthritis, and skin lesions. Less frequent symptoms include gastrointestinal lesions and involvement of the central and peripheral nervous system. The onset of disease is typically in the third or fourth decade of life and equally affects men and women.

BD is common along the ancient Silk Road, which extends from Eastern Asia to the Mediterranean area. The highest prevalence occurs in Turkey (80–420 cases per 100.000), followed by Middle and Far Eastern countries (13.5–20 cases per 100.000). In contrast, the prevalence in Western countries is much lower; ∼0.12–5.1 per 100.000 (1, 2, 3).

The etiology of BD is unknown. It is mainly considered a multi-factorial disease and it is associated with the presence of human leukocyte antigen variants, in particular HLA-B51 (3). Variations in several other genes like the tumor necrosis factor, interleukin 10 (IL10), and the nucleotide-binding oligomerization domain containing two (*NOD2*) genes are also associated with BD (4, 5, 6). Immunohistochemically, BD is characterized by the presence of vasculitis and infiltration of tissue by neutrophils and mononuclear cells (7).

Interestingly, BD also displays some features distinctive of autoimmune processes. First, cellular immunity is disturbed in BD as exemplified by autoreactive T cells-targeting heat shock protein 60 (8). For many autoimmune disorders, autoreactive T cells are a primary culprit in their pathogenesis. Second, several autoantibodies have been described targeting numerous antigens, including CTLA-4 (9) (enhancing T cell proliferation), retinal S-antigen (10), and antikinectin (11), providing evidence for a dysfunctional humoral immune response. In general, BD might be regarded an immune-mediated inflammatory disease.

Glucocorticoids (GC) play a key role in mediating a balanced inflammatory response. GCs exert their effects via interaction with the GC receptor (GR). After binding with its ligand, the GR–GC complex migrates to the nucleus to induce (‘transactivation’) or to suppress (‘transrepression’) expression of target genes. The ultimate biological effects of (endogenous) GC depend on the GC sensitivity of an individual, which is influenced by both genetic and acquired (disease-related) factors. Hence, decreased GC sensitivity could lead to unrestricted immune activation and facilitation of a chronic inflammatory process, a hallmark of many autoimmune disorders.

Indeed, decreased GC sensitivity has been shown to be involved in several autoimmune diseases. For instance, carriers of polymorphisms of the GR gene associated with reduced (i.e. 9β) or increased (i.e. Bcll and N363S) GC sensitivity have increased, respectively, decreased susceptibility to develop rheumatoid arthritis (RA). In addition, carriers of the ER22/23EK allele of the GR gene, which is associated with decreased GC sensitivity, had a more severe disease course (12). At the functional level, van Winsen *et al*. (13) showed that in patients with multiple sclerosis, higher doses of dexamethasone were required to suppress LPS-induced TNF-α production in peripheral blood mononuclear cells (PBMC) when compared with healthy controls. Also in active RA, PBMCs were less sensitive to dexamethasone *in vitro* (14). Finally, an increased expression of the GR splice variant GR-β, the dominant negative inhibitor of the biologically active GR-α, is associated with GC resistance in several inflammatory disorders (15, 16, 17, 18, 19, 20).

As decreased GC sensitivity may contribute to immune-mediated inflammatory diseases, we hypothesized that decreased GC sensitivity is involved in the pathophysiology of BD. To test our hypothesis, we genotyped three independent cohorts of patients with BD for the prevalence of four functional GR polymorphisms. Furthermore, *in vitro* GC sensitivity was assessed by measurement of GR-binding capacity (GR number and affinity) and by two bioassays (21). In these bioassays, dexamethasone-regulated expression of IL2 and GC-induced leucine zipper (GILZ) in PBMC is measured. Transrepressive effects of GC, traditionally considered to be the predominant mechanism regulating anti-inflammatory actions of GC, are represented by the IL2 assay. The GILZ assay embodies all transactivated genes, mediating both anti-inflammatory effects of GC as well as (metabolic) side effects (22, 23). Using these bioassays, a spectrum of GC sensitivity could be demonstrated in healthy individuals (21). Finally, we measured mRNA expression levels of GR-α and GR-β.

## Patients and methods

### Patients

To study the prevalence and distribution of the GR polymorphisms, three cohorts with a total of 290 unrelated BD patients were included in the study (56 patients from the Erasmus MC, Rotterdam; 109 patients from The Jordan Hospital, Amman, Jordan and St John's Ophthalmic Hospital, Jerusalem, Israel; 39 patients from St Thomas' Hospital, London, UK, and 86 patients from the University of Cukurova, Adana, Turkey. Of those, 55 were Caucasians, 125 of Middle Eastern (ME) origin or Arab descent, and 110 patients were of Turkish descent.

The control population consisted of 150 Turkish and 75 ME individuals. Caucasian controls (*n*=5295–5413, depending on polymorphism) were participants in the Rotterdam Study, a population-based prospective cohort study on determinants of disease and disability in persons, aged 55 years and older, living in Rotterdam, The Netherlands.

To study functional differences in *in vitro* GC sensitivity in patients with BD, 19 consecutive BD patients from our outpatient clinic were included in the study. Experienced clinical immunologists (J A M vL and P M vH) examined all patients and assessed disease activity using the validated BD current activity form (BDCAF) (24). As a control group, we studied 20 healthy Caucasian laboratory employees. None of the patients or controls used GCs in the last 3 months. All patients described in this study fulfilled the International Study Group criteria for the diagnosis of BD (25).

### GR polymorphisms

All patients and controls were genotyped for four functional polymorphisms of the GR gene (ER22/23EK, rs6189 and rs6190; N363S, rs6195; Bcll, rs41423247 and 9β, rs6198) (26). DNA was extracted from peripheral venous blood samples using standard techniques. DNA (1–2 ng) was dispensed in 384-well plates. PCR amplification (initial denaturation at 95 °C for 15 min, 40 cycles with denaturation of 15 s at 95 °C, and annealing and extension at 60 °C) and genotyping was performed using the Taqman allelic discrimination assay. Results were analyzed by Taqman Prism 7900HT using the sequence detection system 2.2 software (Applied Biosystems, Nieuwerkerk a/d IJssel, The Netherlands).

### Assessment of in vitro GC sensitivity

#### Functional *in vitro* assays

Recently, two bioassays to determine GC sensitivity were developed in our laboratory (21). In short, peripheral blood was drawn in all patients using Cell Preparation Tubes with sodium heparin (Becton Dickinson, Breda, The Netherlands) allowing isolation of PBMC. Cells were resuspended in RPMI 1640 medium containing l-glutamine supplemented with penicillin (100 U/ml), streptomycin (100 μg/ml), and 10% fetal bovine serum, and precultured overnight in a 48-well plate (Costar, Amsterdam, The Netherlands) at a density of 4.0×10^6^/ml. Trypan blue staining revealed the viability of isolated cells to be >95%. The next day, cells were incubated with increasing doses of dexamethasone (range 0–333 nM) and stimulated with phytohaemagglutinin 10 μg/ml (Sigma–Aldrich). After 4 h in the incubator, total RNA of the cells was collected (total RNA isolation Kit, Roche). cDNA was synthesized using 100 ng RNA and Taqman Reverse Transcription Reagent (N808-0234, Applied Biosystems). For quantitative real-time PCR analysis, the Taqman Technology was applied according to the manufacturer's instructions. GC-specific transactivation of the GILZ mRNA and transrepression of the IL2 mRNA were measured. Half maximal effective concentration (EC_50_) was used as a readout for *in vitro* GC sensitivity. The EC_50_ values of GILZ and IL2 in PBMC were comparable when different compositions of lymphocytes and monocytes were tested (data not shown). In addition, we measured the affinity and number of GR using a whole cell dexamethasone-binding assay, as described previously (27).

#### Gene expression levels of GR isoforms

After isolation of PBMC as described above, 1×10^6^ PMBCs (in duplicate) were lysed and total RNA was extracted immediately (Total RNA isolation Kit, Roche). cDNA was synthesized using 200 ng RNA and Taqman Reverse Transcription Reagent (N808-0234, Applied Biosystems) in a total volume of 50 μl. Gene expression levels of GR-α and GR-β were measured using premanufactured assays (Applied Biosystems, Hs00230818_m1 and Hs00354508_m1 respectively). All results were corrected for the housekeeping gene hypoxanthine phosphoribosyltransferase 1 (*HPRT1*).

### Statistical analysis

To analyze possible associations between GR genotypes and risk of having BD, we calculated odds ratios and 95% confidence intervals for hetero- and homozygous individuals separately (wildtype allele as reference). Given the low number homozygous carriers of the N363S and ER22/23EK minor allele, hetero- and homozygous carriers were analyzed together. Pearson *χ* ^2^-tests were performed to test for differences in distribution of the polymorphisms between the various ethnic groups. Differences in continuous variables between the cohorts were tested using Mann–Whitney *U* tests and ANOVA. IL2-EC_50_ was square-root transformed and number of receptors and *K*_D_ were both natural logarithm transformed to normalize the data. All statistical analysis was performed using SPSS for Windows, release 17.0 (SPSS, Chicago, IL, USA) and we considered differences statistically significant if *P* values were <0.05 (two-sided).

### Ethical approval

This study was approved by the medical ethics committee of the Erasmus Medical Center and all subjects signed informed consent.

## Results

### GR polymorphisms

Healthy controls and BD patients in the three separate cohorts had similar distributions of the four GR polymorphisms (Table 1). Prevalence of the Bcll minor allele was significantly higher in Caucasians compared with both Turkish and ME persons (37.1% in Caucasians vs 21.5 and 21.0% in Turkish and ME persons, respectively, *P*<0.001). In contrast, the 9β minor allele was less prevalent in Caucasians compared with the Turkish and ME persons (17.2% in Caucasians vs 28.5 and 29.6% in Turkish and ME persons, respectively, *P*<0.001).

### In vitro GC sensitivity

We included 19 BD patients from our outpatient clinic. These patients used a wide spectrum of anti-inflammatory agents, including NSAIDs (*n*=7, 36.8%), colchicine (*n*=5, 26.3%), hydroxychloroquine (*n*=4, 21.1%), TNF-α blockers (*n*=2, 10.5%), and pentoxifylline (*n*=3, 15.8%). Thalidomide, methotrexate, interferon-alfa, and octreotide were each used by one patient. Further baseline characteristics and clinical features (present at any time in the disease course) are summarized in Table 2. None of the patients had involvement of the central nervous system.

**Figure 1:**
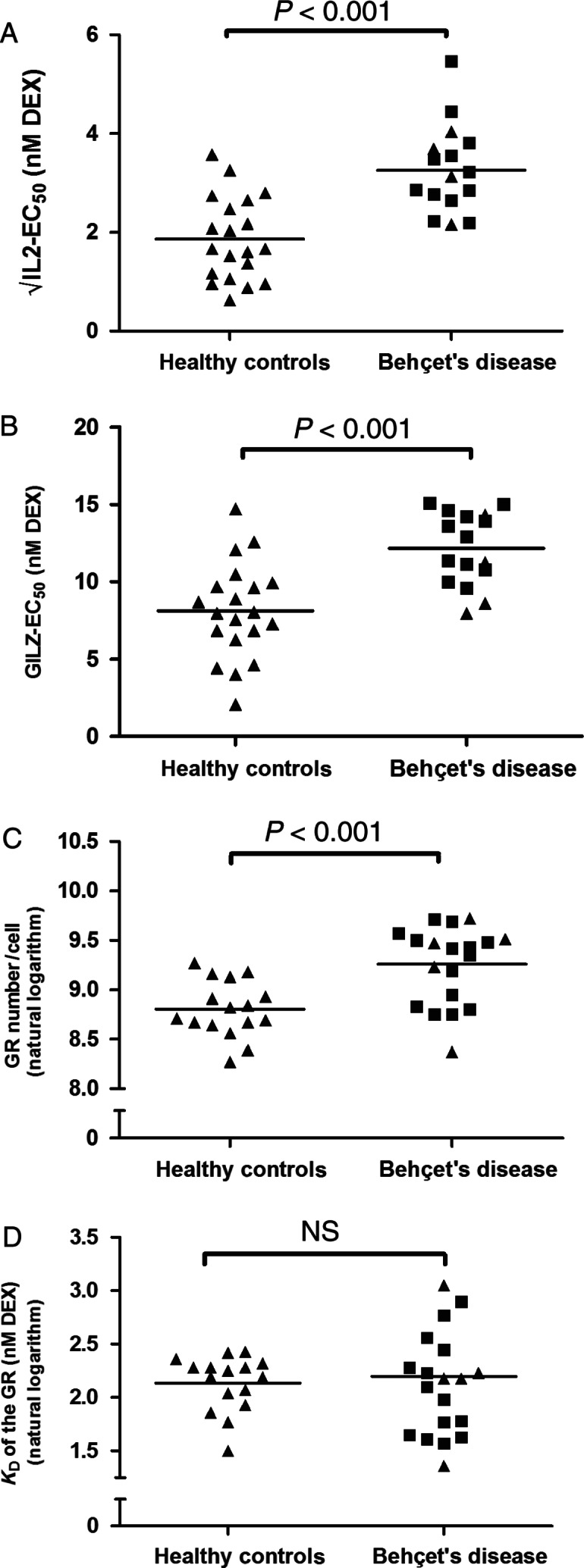
Cellular GC sensitivity in Behçet's disease and healthy controls. EC_50_ (nM DEX) values of interleukin 2 (A) and glucocorticoid (GC)-induced leucine zipper (B) of dexamethasone-treated PBMC. Number of GR per cell and *K*_D_ of the GC receptor using a whole cell-binding assay are depicted in C and D respectively. Triangles (closed triangle) are Caucasian subjects; squares (closed square) represent Turkish–ME subjects. Please note the square-root transformed *Y*-axis in figure (A) and the logarithmically transformed *Y*-axis in figure (C) and (D).

Patients with BD had higher mean EC_50_ values in both the IL2 assay and GILZ assay compared with healthy controls (mean IL2 EC_50_ (95% CI): 10.80 (7.91–14.15) nM in BD vs 3.48 (2.16–5.10) nM in HC, *P*<0.001; mean GILZ EC_50_ (95% CI): 12.16 (10.91–13.42) nM in BD vs 8.13 (6.69–9.58 nM) in HC, *P*<0.001) indicating decreased *in vitro* GC sensitivity in BD (Fig. 1). The maximum induction of GILZ and repression of IL2 did not differ significantly (data not shown). The GR number in PBMC (mean, 95% CI) was higher in BD (10380, 8593–12539 GR/cell) compared with controls (6652, 5719–7738 GR/cell, *P*=0.001), whereas the mean K_D_ (95% CI) of the receptor did not differ between patients (8.34, 6.62–10.50 nM) and controls (8.46, 7.37–9.71 nM). Importantly, the EC_50_ values of GILZ and IL2 and the number of GR did not differ significantly between Caucasian and Turkish and ME patients (Fig. 1). Patients and healthy controls had comparable percentages of monocytes (mean±s.d.: 18.9±5.5 in BD vs 20.9±5.0 in healthy controls). Ligand affinity of monocytes and lymphocytes did not differ significantly. The number of GRs per cell was about three fold higher in monocytes as compared with lymphocytes (data not shown).

No correlations were found between the BDCAF-score and parameters of *in vitro* GC sensitivity. Men and women had equal mean levels of IL2-EC_50_ and GILZ-EC_50_. Likewise, there were no gender differences at the level of the number of GR or the affinity of the GR.

**Figure 2:**
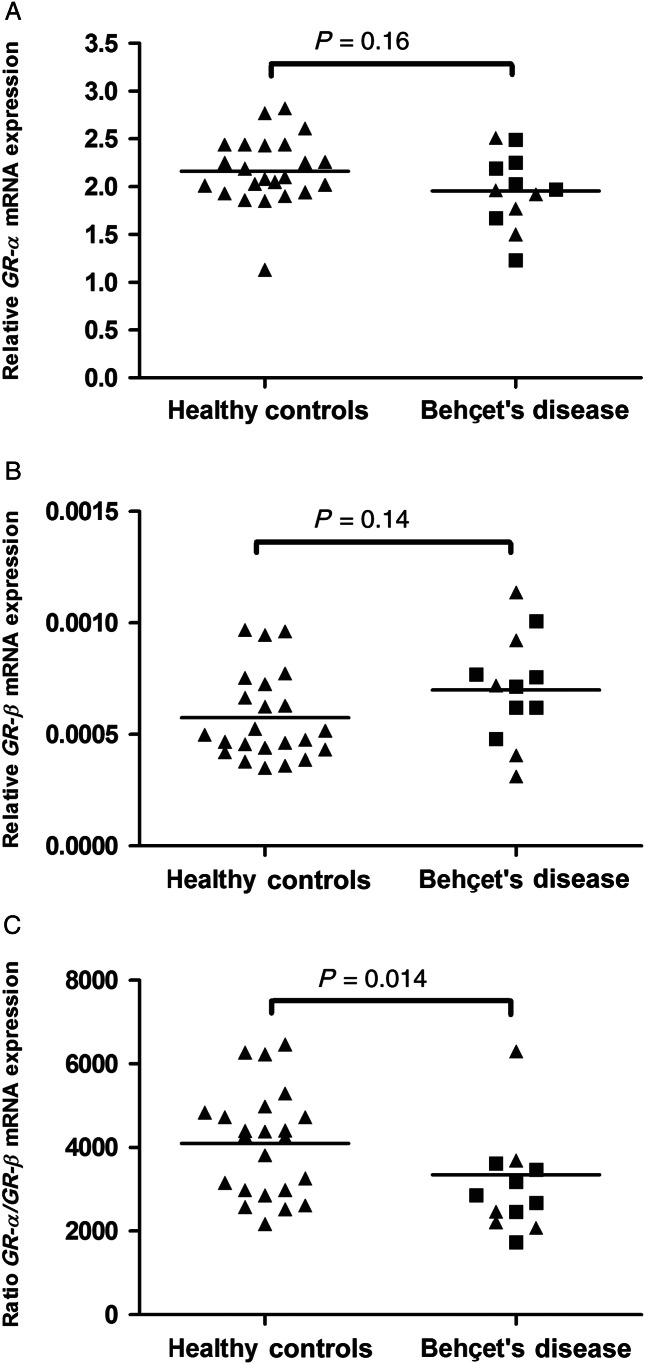
mRNA expression levels of the glucocorticoid receptor isoforms in PBMC of patients with Behçet's disease and healthy controls. GR-α is the biologically active isoform (A), GR-β acts as a dominant negative isoform (B). The ratio of GR-α/GR-β is shown in C. Triangles (closed triangle) are Caucasian subjects; squares (closed square) represent Turkish–ME subjects.

In 12 BD patients and healthy controls, mRNA expression levels of GR-α and GR-β were measured. BD patients showed a trend toward lower mRNA expression levels of GR-α whereas mRNA expression levels of GR-β tended to be higher. Combined, the GR-α/GR-β ratio was significantly lower in patients (*P*=0.014; Fig. 2).

## Discussion

The results of our study suggest that decreased GC sensitivity might play a role in the pathophysiology of BD. More specifically, both transactivating and transrepressing pathways of GC action seem to be affected in BD, together with an altered expression of the GR in PBMC. At the transcriptional level, a lower GR-α/GR-β ratio was observed in BD.

We examined the prevalence of four functional GR polymorphisms in three independent cohorts. None of the GR polymorphisms was associated with susceptibility to BD, consistent with two recent genome-wide association studies from Turkey and Japan (5, 28). However, we found significant differences in the prevalence of GR polymorphisms between the Caucasian and Turkish and ME cohort, which have not been reported before. The BclI minor allele is associated with increased GC sensitivity (26) and is present at a lower frequency in the Turkish–ME cohort. The Bcll minor allele is also less prevalent in other areas with relatively high prevalence of BD (e.g. China, Korea), as compared with allele frequencies observed in Caucasian populations (29, 30). On the other hand, the 9β minor allele, which is associated with decreased GC sensitivity (26), showed a lower prevalence in the Caucasian population. The clinical relevance of these observations is yet unclear, but they do not directly support the concept of a ‘GC-resistant’ genetic profile contributing to the development of BD as a comparable prevalence of the Bcll and 9β minor alleles was found in the Turkish and ME patients and healthy controls. Future studies should examine whether the different prevalence pattern of GR polymorphisms in the Turkish–ME population is associated with other immune-mediated disorders.

To further explore the role of GC sensitivity in BD, we assessed transactivating and transrepressing capacity of GC *in vitro* by measuring the EC_50_ values of two representative GC-mediated genes, *GILZ* (*TSC22D3*) and *IL2*. We measured higher EC_50_ values of both genes in BD, indicating decreased *in vitro* GC sensitivity compared with healthy controls. In contrast, we found higher numbers of GR per cell, which might reflect a compensatory upregulation of the GR. Importantly, most patients in our study had relatively low BDCAF scores, suggesting that the higher EC_50_ values are not solely influenced by higher levels of pro-inflammatory cytokines, a well-known mechanism of acquired GC resistance (31). Diminished (counterbalancing) cellular effects of GC on the immune system could allow for the development of chronic (auto)-inflammatory processes as in BD. A point of future attention is that in this relatively small group of patients there was considerable variation with respect to the use/not use of disease-modifying drugs. In this setting, it was not possible to analyze the possible effects of these drugs on the outcome of the assays.

In clinical practice, GCs are widely used in BD. However, the only randomized clinical trial studying the effects of GC in BD showed a lack of efficacy of GC treatment. In this study, 3-weekly depots of 40 mg methylprednisolone acetate during 27 weeks in patients with active BD demonstrated no benefit over placebo-treated patients with respect to orogenital ulcers, folliculitis, and arthritis, although lesions with erythema nodosum did improve following GC treatment (32). Interestingly, in asthma and RA approximately one-third of patients are also GC resistant (33, 34). In addition, a case series reported by Tanaka *et al*. (35) showed that patients who had ocular manifestations of BD with low *in vitro* GC sensitivity had a worse clinical course as defined by more frequent relapses of ocular inflammation and higher intra-ocular pressure. Therefore, the observed decreased *in vitro* GC sensitivity in BD may not only contribute to an increased understanding of the (etio) pathophysiology of the disease but could also have direct clinical implications. Obviously, it would be of great interest to study whether assessment of *in vitro* GC sensitivity, as measured by the IL2, GILZ, and whole cell dexamethasone-binding assays, correlates with *in vivo* response to GC therapy in BD. Insights in the patients response to exogenously administered GC before the start of therapy could then be used to facilitate more individualized GC therapy.

In order to examine possible mechanisms underlying this decreased GC sensitivity in BD, we determined mRNA expression levels of the α and β splice variant of the GR. GR-β is thought to act as a dominant negative inhibitor of the biologically active GR-α by means of competition for co-factors, formation of inactive heterodimers with GR-α, and possibly competition for GRE *in vitro* (15, 16, 19). High expression of GR-β *in vivo* has been associated with GC-resistant states in inflammatory bowel disease, asthma, and RA (17, 18, 20, 36, 37). In our cohort, the ratio of GR-α/GR-β was significantly lower in patients with BD and could therefore partially explain the decreased cellular GC sensitivity in BD, although the clinical relevance of the very low expression levels of GR-β are still the subject of debate. Other mechanisms possibly underlying the decreased cellular GC sensitivity in BD may include disturbed nuclear trafficking of the GR via phosphomodulation by kinases and phosphatases, interference with the transcriptional machinery by histone acetyltransferases modulating protein acetylation or transcriptional blocking by altered expression of micro-RNAs.

It must be kept in mind that our data represent relatively small groups, with mixed ethnic background. In this perspective, it is important to note that the Caucasian and Turkish–ME patients with BD were equally distributed with respect to the bioassays, GR assay, and the gene expression levels. Therefore, we assumed that ethnic background is not a major factor determining outcomes of the bioassay, GR assay or gene expression levels, and analyzed Caucasian and Turkish–ME patients together. Also, mRNA levels of GR-α and GR-β do not necessarily represent protein expression levels in our patients. Finally, the interpretation of cross-sectional data is limited with respect to dynamic processes as the pathogenesis of BD. Therefore, longitudinal studies evaluating GC sensitivity at various stages of BD, including recent-onset disease, and different levels of disease activity will provide more insight into the importance of GC sensitivity and the development of BD.

In conclusion, polymorphisms of the GR gene are not associated with susceptibility to BD. However, our *in vitro* data indicate decreased cellular GC sensitivity in BD. This altered GC sensitivity could play an as yet unidentified role in the etiopathophysiology of BD. A decreased GR-α/GR-β ratio may in part explain this decreased GC sensitivity.

## Figures and Tables

**Table 1:** Frequencies of GR polymorphisms in Behçet's disease and healthy controls.

**Polymorphism**	**Caucasian group**			**Turkish group**			**Mid-East group**		
Case, *n* (%)	Control, *n* (%)	OR (95% CI)	Case, *n* (%)	Control, *n* (%)	OR (95% CI)	Case, *n* (%)	Control, *n* (%)	OR (95% CI)
ER22/23EK									
Noncarriers	55 (100)	4959 (93.7)	Reference	104 (94.5)	144 (97.3)	Reference	124 (99.2)	73 (97.3)	Reference
Carriers	–	336 (6.4)^a^	NA	6 (5.5)^b^	4 (2.7)^b^	2.07 (0.57–7.55)	1 (0.8)^b^	2 (2.7)^b^	0.29 (0.26–3.30)
N363S									
Noncarriers	52 (94.5)	4932 (92.7)	Reference	107 (97.3)	147 (98.7)	Reference	124 (99.2)	75 (100)	Reference
Carriers	3 (5.5)^b^	388 (7.3)^c^	0.74 (0.23–2.39)	3 (2.7)^b^	2 (1.3)^b^	2.06 (0.34–12.55)	1 (0.8)^b^	–	NA
BclI									
Noncarriers	25 (46.3)	2133 (39.4)	Reference	67 (60.9)	89 (60.1)	Reference	76 (60.8)	50 (66.7)	Reference
Carriers	29 (53.7)	3280 (60.6)	0.75 (0.44–1.29)	43 (39.1)	59 (39.9)	0.97 (0.58–1.60)	49 (39.2)	25 (33.3)	1.29 (0.71–2.35)
Heterozygous carriers	23 (42.6)	2539 (46.9)	0.77 (0.43–1.36)	40 (36.4)	53 (35.8)	1.00 (0.60–1.68)	43 (34.4)	21 (28.0)	1.35 (0.71–2.54)
Homozygous carriers	6 (11.1)	741 (13.7)	0.69 (0.28–1.69)	3 (2.7)	6 (4.1)	0.66 (0.16–2.75)	6 (4.8)	4 (5.3)	0.99 (0.27–3.67)
9β									
Noncarriers	38 (71.7)	3681 (71.4)	Reference	53 (50.5)	76 (51.4)	Reference	62 (52.1)	33 (44.0)	Reference
Carriers	15 (28.3)	1692 (28.6)	0.86 (0.47–1.57)	52 (49.5)	72 (48.6)	1.04 (0.63–1.71)	57 (47.9)	42 (56.0)	0.72 (0.40–1.29)
Heterozygous carriers	13 (24.5)	1531 (24.5)	0.82 (0.44–1.55)	41 (39)	63 (42.6)	0.93 (0.55–1.58)	49 (41.2)	34 (45.3)	0.77 (0.42–1.41)
Homozygous carriers	2 (3.8)	161 (4.1)	1.20 (0.29–5.00)	11 (10.5)	9 (6.0)	1.75 (0.68–4.52)	8 (6.7)	8 (10.7)	0.53 (0.18–1.55)

NA, not applicable (zero cases in one of the groups); OR, odds ratio.

a Eight homozygous carriers.

b All heterozygous carriers.

c Five homozygous carriers.

**Table 2:** Patient characteristics. Values are presented as number (%), unless otherwise stated.

	**Healthy controls** (*n*=20)	**Behçet's disease** (*n*=19)
Female gender	10 (50)	12 (63.2)
Age (years), mean (s.d.)	31.8 (9.7)	43.3 (10.6)
Caucasian ethnicity	20 (100)	5 (26.3)
BDCAF, median (range)	–	12 (0–30)
Phenotype of disease (ever)		
Oral ulcers	–	19 (100)
Genital ulcers	–	17 (89.5)
Arthralgia/arthritis	–	15 (78.9)
Gastrointestinal involvement	–	15 (78.9)
Uveitis/vasculitis retinae	–	11 (57.9)
Positive pathergy test	–	4 (21.1)^a^
Erythema nodosum	–	11 (57.9)
Pustulopapular skin lesions	–	15 (78.9)

BDCAF, Behçet's disease current activity form.

a Three patients never underwent a pathergy test.

## References

[1] Azizlerli G, Kose AA, Sarica R, Gul A, Tutkun IT, Kulac M, Tunc R, Urgancioglu M, Disci R (2003). Prevalence of Behcet's disease in Istanbul, Turkey. International Journal of Dermatology.

[2] Grana J, Sanchez-Meizoso MO, Galdo F (2001). Epidemiological aspects of Behcet's disease in Galicia. Journal of Rheumatology.

[3] Sakane T, Takeno M, Suzuki N, Inaba G (1999). Behcet's disease. New England Journal of Medicine.

[4] Kappen JH, Wallace GR, Stolk L, Rivadeneira F, Uitterlinden AG, van Daele PL, Laman JD, Kuijpers RW, Baarsma GS, Stanford MR, Fortune F, Madanat W, van Hagen PM, van Laar JA (2009). Low prevalence of NOD2 SNPs in Behcet's disease suggests protective association in Caucasians. Rheumatology.

[5] Remmers EF, Cosan F, Kirino Y, Ombrello MJ, Abaci N, Satorius C, Le JM, Yang B, Korman BD, Cakiris A, Aglar O, Emrence Z, Azakli H, Ustek D, Tugal-Tutkun I, Akman-Demir G, Chen W, Amos CI, Dizon MB, Kose AA, Azizlerli G, Erer B, Brand OJ, Kaklamani VG, Kaklamanis P, Ben-Chetrit E, Stanford M, Fortune F, Ghabra M, Ollier WE, Cho YH, Bang D, O'Shea J, Wallace GR, Gadina M, Kastner DL, Gül A (2010). Genome-wide association study identifies variants in the MHC class I, IL10, and IL23R-IL12RB2 regions associated with Behcet's disease. Nature Genetics.

[6] Touma Z, Farra C, Hamdan A, Shamseddeen W, Uthman I, Hourani H, Arayssi T (2010). TNF polymorphisms in patients with Behcet disease: a meta-analysis. Archives of Medical Research.

[7] Canete JD, Celis R, Noordenbos T, Moll C, Gomez-Puerta JA, Pizcueta P, Palacin A, Tak PP, Sanmarti R, Baeten D (2009). Distinct synovial immunopathology in Behcet disease and psoriatic arthritis. Arthritis Research & Therapy.

[8] Direskeneli H, Saruhan-Direskeneli G (2003). The role of heat shock proteins in Behcet's disease. Clinical and Experimental Rheumatology.

[9] Matsui T, Kurokawa M, Kobata T, Oki S, Azuma M, Tohma S, Inoue T, Yamamoto K, Nishioka K, Kato T (1999). Autoantibodies to T cell costimulatory molecules in systemic autoimmune diseases. Journal of Immunology.

[10] Kurhan-Yavuz S, Direskeneli H, Bozkurt N, Ozyazgan Y, Bavbek T, Kazokoglu H, Eksioglu-Demiralp E, Wildner G, Diedrichs-Mohring M, Akoglu T (2000). Anti-MHC autoimmunity in Behcet's disease: T cell responses to an HLA-B-derived peptide cross-reactive with retinal-S antigen in patients with uveitis. Clinical and Experimental Immunology.

[11] Feng XG, Ye S, Lu Y, Xu XJ, Gu YY, Shen N, Ye P, Cheng FP, Wang AM, Chen SL (2007). Antikinectin autoantibody in Behcet's disease and several other autoimmune connective tissue diseases. Clinical and Experimental Rheumatology.

[12] van Oosten MJ, Dolhain RJ, Koper JW, van Rossum EF, Emonts M, Han KH, Wouters JM, Hazes JM, Lamberts SW, Feelders RA (2010). Polymorphisms in the glucocorticoid receptor gene that modulate glucocorticoid sensitivity are associated with rheumatoid arthritis. Arthritis Research & Therapy.

[13] van Winsen LM, Muris DF, Polman CH, Dijkstra CD, van den Berg TK, Uitdehaag BM (2005). Sensitivity to glucocorticoids is decreased in relapsing remitting multiple sclerosis. Journal of Clinical Endocrinology and Metabolism.

[14] Quax RA, Koper JW, de Jong PH, van Heerebeek R, Weel AE, Huisman AM, van Zeben D, de Jong FH, Lamberts SW, Hazes JM, Feelders RA (2012). *In vitro* glucocorticoid sensitivity is associated with clinical glucocorticoid therapy outcome in rheumatoid arthritis. Arthritis Research & Therapy.

[15] Bamberger CM, Bamberger AM, de Castro M, Chrousos GP (1995). Glucocorticoid receptor β, a potential endogenous inhibitor of glucocorticoid action in humans. Journal of Clinical Investigation.

[16] Charmandari E, Chrousos GP, Ichijo T, Bhattacharyya N, Vottero A, Souvatzoglou E, Kino T (2005). The human glucocorticoid receptor (hGR) β isoform suppresses the transcriptional activity of hGRalpha by interfering with formation of active coactivator complexes. Molecular Endocrinology.

[17] Fujishima S, Takeda H, Kawata S, Yamakawa M (2009). The relationship between the expression of the glucocorticoid receptor in biopsied colonic mucosa and the glucocorticoid responsiveness of ulcerative colitis patients. Clinical Immunology.

[18] Honda M, Orii F, Ayabe T, Imai S, Ashida T, Obara T, Kohgo Y (2000). Expression of glucocorticoid receptor β in lymphocytes of patients with glucocorticoid-resistant ulcerative colitis. Gastroenterology.

[19] Oakley RH, Jewell CM, Yudt MR, Bofetiado DM, Cidlowski JA (1999). The dominant negative activity of the human glucocorticoid receptor β isoform. Specificity and mechanisms of action. Journal of Biological Chemistry.

[20] Sousa AR, Lane SJ, Cidlowski JA, Staynov DZ, Lee TH (2000). Glucocorticoid resistance in asthma is associated with elevated *in vivo* expression of the glucocorticoid receptor β-isoform. Journal of Allergy and Clinical Immunology.

[21] Smit P, Russcher H, de Jong FH, Brinkmann AO, Lamberts SW, Koper JW (2005). Differential regulation of synthetic glucocorticoids on gene expression levels of glucocorticoid-induced leucine zipper and interleukin-2. Journal of Clinical Endocrinology and Metabolism.

[22] Clark AR (2007). Anti-inflammatory functions of glucocorticoid-induced genes. Molecular and Cellular Endocrinology.

[23] Schacke H, Docke WD, Asadullah K (2002). Mechanisms involved in the side effects of glucocorticoids. Pharmacology & Therapeutics.

[24] Bhakta BB, Brennan P, James TE, Chamberlain MA, Noble BA, Silman AJ (1999). Behcet's disease: evaluation of a new instrument to measure clinical activity. Rheumatology.

[25] International Study Group for Behçet's Disease. Criteria for diagnosis of Behcet's disease. *Lancet* 1990 **335** 1078–1080. 10.1016/0140-6736(90)92643-V 1970380

[26] Manenschijn L, van den Akker EL, Lamberts SW, van Rossum EF (2009). Clinical features associated with glucocorticoid receptor polymorphisms. An overview. Annals of the New York Academy of Sciences.

[27] Molijn GJ, Spek JJ, van Uffelen JC, de Jong FH, Brinkmann AO, Bruining HA, Lamberts SW, Koper JW (1995). Differential adaptation of glucocorticoid sensitivity of peripheral blood mononuclear leukocytes in patients with sepsis or septic shock. Journal of Clinical Endocrinology and Metabolism.

[28] Mizuki N, Meguro A, Ota M, Ohno S, Shiota T, Kawagoe T, Ito N, Kera J, Okada E, Yatsu K, Song YW, Lee EB, Kitaichi N, Namba K, Horie Y, Takeno M, Sugita S, Mochizuki M, Bahram S, Ishigatsubo Y, Inoko H (2010). Genome-wide association studies identify IL23R-IL12RB2 and IL10 as Behcet's disease susceptibility loci. Nature Genetics.

[29] Duan ZX, Gu W, Du DY, Hu P, Jiang DP, Zhu PF, Wang ZG, Jiang JX (2009). Distributions of glucocorticoid receptor gene polymorphisms in a Chinese Han population and associations with outcome after major trauma. Injury.

[30] Lee EB, Kim JY, Lee YJ, Song YW (2005). Glucocorticoid receptor polymorphisms in Korean patients with rheumatoid arthritis. Annals of Rheumatic Disease.

[31] Schaaf MJ, Cidlowski JA (2002). Molecular mechanisms of glucocorticoid action and resistance. Journal of Steroid Biochemistry and Molecular Biology.

[32] Mat C, Yurdakul S, Uysal S, Gogus F, Ozyazgan Y, Uysal O, Fresko I, Yazici H (2006). A double-blind trial of depot corticosteroids in Behcet's syndrome. Rheumatology.

[33] Barnes PJ, Adcock IM (2009). Glucocorticoid resistance in inflammatory diseases. Lancet.

[34] Silverman MN, Sternberg EM (2008). Neuroendocrine–immune interactions in rheumatoid arthritis: mechanisms of glucocorticoid resistance. Neuroimmunomodulation.

[35] Tanaka T, Suzuki J, Yamakawa N, Usui M (2000). Steroid sensitivity and postoperative course of seven patients with Behcet's disease. Ophthalmic Research.

[36] Chikanza IC (2002). Mechanisms of corticosteroid resistance in rheumatoid arthritis: a putative role for the corticosteroid receptor β isoform. Annals of the New York Academy of Sciences.

[37] Goecke A, Guerrero J (2006). Glucocorticoid receptor β in acute and chronic inflammatory conditions: clinical implications. Immunobiology.

